# Moxetumomab pasudotox in heavily pre-treated patients with relapsed/refractory hairy cell leukemia (HCL): long-term follow-up from the pivotal trial

**DOI:** 10.1186/s13045-020-01004-y

**Published:** 2021-02-24

**Authors:** Robert J. Kreitman, Claire Dearden, Pier Luigi Zinzani, Julio Delgado, Tadeusz Robak, Philipp D. le Coutre, Bjørn T. Gjertsen, Xavier Troussard, Gail J. Roboz, Lionel Karlin, Douglas E. Gladstone, Nataliya Kuptsova-Clarkson, Shiyao Liu, Priti Patel, Federico Rotolo, Emmanuel Mitry, Ira Pastan, Francis Giles, the Study 1053 investigators

**Affiliations:** 1 Clinical Immunotherapy Section, Laboratory of Molecular Biology, National Cancer Institute, National Institutes of Health, 9000 Rockville Pike, Bethesda, MD 20892 USA; 2 The Royal Marsden Hospital, Downs Road, Sutton, England, UK; 3 Azienda Ospedaliero-Universitaria di Bologna, Via Albertoni 15, Bologna, Italia; 4 Istituto di Ematologia, “Seràgnoli”, Dipartimento di Medicina Specialistica, Diagnostica e Sperimentale Università Degli Studi, Bologna, Italia; 5 Hospital Clinic Barcelona, IDIBAPS, Barcelona, Spain; 6 Medical University of Łódź and Copernicus Memorial Hospital, Pabianicka 62, 90-001 Łódź, Poland; 7 Charité – Universitätsmedizin Berlin, Charitépl. 1, 10117 Berlin, Germany; 8 Haukeland University Hospital and University of Bergen, Jonas Lies vei 65, 5021 Bergen, Norway; 9 Hospital Center University of Caen Normandie, Avenue de La Côte de Nacre, 14000 Caen, France; 10 Weill Cornell Medical College, The New York Presbyterian Hospital, 525 E 68th St, New York, NY USA; 11 Hôpital Lyon Sud, Hospices Civils de Lyon, 165 Chemin du Grand Revoyet, 69310 Pierre-Bénite, Lyon, France; 12 Sidney Kimmel Comprehensive Cancer Center, Johns Hopkins University, 401 N Broadway, Baltimore, MD USA; 13 AstraZeneca, One MedImmune Way, Gaithersburg, MD USA; 14 Acerta Pharma (AstraZeneca), 121 Oyster Point Blvd, South San Francisco, CA USA; 15 Innate Pharma, 117 Avenue de Luminy, BP 30191, 13276 Marseille, France; 16 Developmental Therapeutics Consortium, 175 E Delaware Pl #7204, Chicago, IL USA

**Keywords:** Hairy cell leukemia (HCL), B cell malignancy, Relapsed/refractory, CD22, Immunotoxin, Moxetumomab pasudotox, Minimal residual disease (MRD)

## Abstract

**Background:**

Moxetumomab pasudotox is a recombinant CD22-targeting immunotoxin. Here, we present the long-term follow-up analysis of the pivotal, multicenter, open-label trial (NCT01829711) of moxetumomab pasudotox in patients with relapsed/refractory (R/R) hairy cell leukemia (HCL).

**Methods:**

Eligible patients had received ≥ 2 prior systemic therapies, including ≥ 2 purine nucleoside analogs (PNAs), or ≥ 1 PNA followed by rituximab or a BRAF inhibitor. Patients received 40 µg/kg moxetumomab pasudotox intravenously on Days 1, 3, and 5 of each 28-day cycle for up to six cycles. Disease response and minimal residual disease (MRD) status were determined by blinded independent central review. The primary endpoint was durable complete response (CR), defined as achieving CR with hematologic remission (HR, blood counts for CR) lasting > 180 days.

**Results:**

Eighty adult patients were treated with moxetumomab pasudotox and 63% completed six cycles. Patients had received a median of three lines of prior systemic therapy; 49% were PNA-refractory, and 38% were unfit for PNA retreatment. At a median follow-up of 24.6 months, the durable CR rate (CR with HR > 180 days) was 36% (29 patients; 95% confidence interval: 26–48%); CR with HR ≥ 360 days was 33%, and overall CR was 41%. Twenty-seven complete responders (82%) were MRD-negative (34% of all patients). CR lasting ≥ 60 months was 61%, and the median progression-free survival without the loss of HR was 71.7 months. Hemolytic uremic and capillary leak syndromes were each reported in ≤ 10% of patients, and ≤ 5% had grade 3–4 events; these events were generally reversible. No treatment-related deaths were reported.

**Conclusions:**

Moxetumomab pasudotox resulted in a high rate of durable responses and MRD negativity in heavily pre-treated patients with HCL, with a manageable safety profile. Thus, it represents a new and viable treatment option for patients with R/R HCL, who currently lack adequate therapy.

***Trial registration*:**

ClinicalTrials.gov identifier: NCT01829711; first submitted: April 9, 2013. https://clinicaltrials.gov/ct2/show/NCT01829711

**Supplementary information:**

**Supplementary information** accompanies this paper at 10.1186/s13045-020-01004-y.

## Background

Hairy cell leukemia (HCL) is a rare mature B-cell malignancy with high CD22 expression [1]. The mainstay of therapy and recommended first-line treatment is a purine nucleoside analog (PNA) such as pentostatin or cladribine [2–4]. Although the majority of patients achieve long-term remission with PNAs, eventual disease relapse and diminishing clinical outcomes in later lines exemplify an important unmet need for therapies in relapsed/refractory (R/R) HCL [4–7]. Novel targeted therapies such as rituximab (anti-CD20 monoclonal antibody), ibrutinib (Bruton tyrosine kinase inhibitor), and vemurafenib (BRAF inhibitor) have demonstrated some efficacy in pre-treated patients but have not yet been rigorously evaluated in robust studies with long-term follow-up, nor approved for HCL treatment [8–20].

Notably, the current treatment options are frequently associated with safety and tolerability concerns. PNA therapy is immunosuppressive and thus associated with opportunistic infections and second malignancies in already vulnerable patients [2, 4, 5, 21]. Cladribine, in particular, is not recommended in those with an active infection, and patients must be closely monitored for symptoms of infection [2]. Rituximab can also lead to severe infections, along with thrombocytopenia, and vemurafenib is associated with cutaneous conditions including photosensitivity and skin papillomas [8, 9, 11, 13, 15].

One growing area of interest with relevance to the treatment of HCL is the concept of minimal residual disease (MRD), defined as the detection of tumor cells below the sensitivity level of conventional cytomorphology using techniques such as cytogenetics, flow cytometry, PCR, and high-throughput sequencing [22, 23]. MRD is widely used as a measure of tumor burden for other hematologic malignancies, serving as a significant prognostic factor for both acute lymphoblastic leukemia and chronic myeloid leukemia and informing treatment decisions [22, 24, 25]. In HCL, the implications of MRD remain unclear; some studies report that MRD positivity after PNA therapy is predictive for disease relapse, suggesting that MRD eradication may improve clinical outcomes [26, 27]. Current treatments, including targeted therapies, are unable to completely eradicate MRD in all patients with R/R HCL [8–19].

In September 2018, moxetumomab pasudotox (CAT-8015), a first-in-class recombinant CD22-directed cytotoxin [28], was approved for the treatment of adult patients with R/R HCL in the USA. The pivotal multicenter, open-label, single-arm trial (NCT01829711) evaluated the efficacy, safety, immunogenicity, and pharmacokinetics of moxetumomab monotherapy in patients with R/R HCL who had previously received ≥ 2 systemic therapies, including ≥ 1 PNA. To date, this is the largest study in heavily pre-treated HCL (*N* = 80). It was the first study to evaluate the efficacy and safety of a third-line treatment with long-term follow-up and to report durable complete response (CR) as a primary endpoint. Moreover, it was also one of few studies to prospectively assess MRD status after therapy in HCL.

At a median follow-up of 16.7 months (range: 2.1–48.8 months), the primary analysis showed a durable CR rate of 30% (24 patients), CR rate of 41% (33 patients), and MRD negativity in 82% (27 patients) of complete responders. Furthermore, moxetumomab pasudotox demonstrated acceptable safety and manageable tolerability [29]. In this report, we present the final analysis describing the long-term follow-up of efficacy and safety of moxetumomab pasudotox in patients with R/R HCL, with a median follow-up of 24.6 months (range: 1.2–71.7 months).

## Methods

### Study design and patient eligibility

This multicenter, open-label, single-arm study enrolled patients from 32 centers across 14 countries. Adults (aged ≥ 18 years) with histologically confirmed HCL or variant HCL and an indication for therapy were eligible for this study. An indication for therapy was defined as one or more of the following: neutrophils < 1.0 × 10^3^/µL, platelets < 100 × 10^3^/µL, hemoglobin < 10 g/dL, or symptomatic splenomegaly. Patients must have been treated with a minimum of two systemic therapies, including two courses of a PNA, or one course of a PNA followed by rituximab or a BRAF inhibitor. Patients must not have had prior treatment with moxetumomab pasudotox and were required to have an Eastern Cooperative Oncology Group (ECOG) performance status ≤ 2. Patients with impaired renal or hepatic function, active second malignancies requiring treatment, and uncontrolled intercurrent illnesses such as an ongoing or active infection were excluded. Detailed inclusion and exclusion criteria can be found in the study protocol.

### Study treatment

Moxetumomab pasudotox was administered at a dose of 40 µg/kg by intravenous (i.v.) infusion over 30 min on Days 1, 3, and 5 of a 28-day cycle for up to six cycles, or until documentation of MRD-negative CR, disease progression, initiation of an alternate therapy, or unacceptable toxicity.

If during treatment and prior to completion of 6 cycles of therapy, blood counts were consistent with CR for at least 4 weeks, an interim disease assessment could have been performed at the discretion of the investigator. If a CR without MRD was documented, treatment was discontinued.

Patients were given prophylactic treatments for renal insufficiency (fluids and aspirin), arterial thrombosis (low-dose aspirin), and hypersensitivity reactions (hydroxyzine, acetaminophen, and ranitidine). Details can be found in the study protocol. Patients were encouraged to drink at least 3 L of fluid per day on Days 0–8 of the treatment cycle.

Adequate pneumocystis carinii pneumonia prophylaxis was provided for patients receiving corticosteroids ≥ 10 mg of prednisone daily or equivalent, CD4 lymphocyte count < 0.2 × 10^3^/µL, or at the discretion of the investigator. Viral prophylaxis was provided for patients receiving chronic corticosteroids or with lymphopenia.

### Study endpoints

The primary endpoint was the durable CR rate, defined as CR with hematologic remission (HR) lasting > 180 days; it was calculated as the ratio of the number of patients achieving durable CR over the total number of patients treated. CR was determined by blinded independent central review (BICR) and local investigators based on pathology (no evidence of leukemic cells in both the peripheral blood and bone marrow by routine hematoxylin and eosin [H&E] staining), radiography with CT or MRI (no hepatomegaly, splenomegaly, or lymphadenopathy) and HR (neutrophils ≥ 1.5 × 10^3^/µL, platelets ≥ 100 × 10^3^/µL, and hemoglobin ≥ 11.0 g/dL, without transfusions or growth factors for ≥ 4 weeks). Resolution of splenomegaly required a maximum spleen diameter of < 17 cm or > 25% shorter than at baseline. Relapse was defined as the loss of any criteria needed for best response, including asymptomatic reappearance of hairy cells in the bone marrow by H&E staining. Additional details can be found in the study protocol.

The secondary endpoints included CR rate, objective response (OR) rate, time to and duration of CR and OR, MRD, progression-free survival (PFS) rates, safety/tolerability, immunogenicity, and pharmacokinetics.

MRD was locally assessed during treatment using quantitative flow cytometric analysis of peripheral blood samples or bone marrow aspirate, according to the institution’s protocols, and by immunohistochemistry (IHC) on the bone marrow biopsy tissue. Local study sites received instructions on sample collection and submission for analysis. Samples for peripheral blood were collected at screening, prior to Cycles 3 and 5 only, at the end of treatment, at 181 days after end of treatment, 12 and 18 months after end of treatment, and yearly thereafter. Bone marrow aspirate was collected at screening, at the end of treatment for all patients, and 181 days after end of treatment for patients in PR or CR. Local MRD status was then confirmed by BICR evaluation of H&E staining and IHC on the bone marrow biopsy tissue. IHC involved staining for the presence of phenotypical B-cell (CD20) and HCL (CD79a, annexin A1, DBA.44, and PAX-5) antigens. MRD negativity was defined as the absence of HCL phenotype cells in the bone marrow biopsy by IHC and in the bone marrow aspirate/blood by flow cytometry.

Safety was evaluated from enrollment until 30 days after the last moxetumomab pasudotox dose was administered. The evaluation included adverse events (AEs), serious AEs (SAEs), vital signs, electrocardiograms, physical examinations, and clinically meaningful changes in laboratory tests. The National Cancer Institute Common Terminology Criteria v4.03 were used for grading, and the Medical Dictionary for Regulatory Activities v20.0 was used for coding AEs.

### Statistical analysis

The intent-to-treat (ITT) population, including all patients who received moxetumomab pasudotox, was used to evaluate efficacy, whereas the safety population, including all patients who received ≥ 1 dose of moxetumomab pasudotox, was used to evaluate safety. Both populations included the same 80 patients.

The sample size was based on the historical CR rate of rituximab against relapsed HCL requiring treatment of ≤ 13% [11] and the target durable CR for moxetumomab pasudotox of ≥ 28%. Using the exact binomial test, it was estimated that a sample size of 77 patients would provide 90% power to detect a difference between 13 and 28% durable CR rates, with a two-sided significance level of 0.05. The 95% confidence interval (CI) for durable CR was estimated using the exact probability method (Clopper–Pearson exact interval); if the lower bound of the 95% CI was above 13%, the durable CR rate was deemed significantly higher than the historical control. The Kaplan–Meier method was used to estimate the duration of CR, OR, and PFS. In these analyses, patients alive with no documented relapse prior to data cutoff, dropout, or initiation of an alternate therapy, were censored on the last date of disease or hematologic assessment.

## Results

### Patients

Eighty patients were enrolled and treated with moxetumomab pasudotox (Table 1); the first patient received the first dose of moxetumomab pasudotox on May 2, 2013, and the last patient received the last dose on May 30, 2016 (data cutoff on April 29, 2019). Patients had received a median of three lines of prior systemic therapy. Thirty-nine (49%) patients had PNA-refractory disease, and 30 (38%) patients were unfit for PNA re-treatment (Table 1). Among these 30 patients, 20 patients had grade 4 neutropenia at baseline and 19 patients had a significant ongoing infection in their medical history (i.e., cellulitis, diverticulitis, hepatitis B virus, herpes zoster, sinusitis, and wound infection) and/or experienced a grade ≥ 3 infection (i.e., pneumonia and sepsis) or febrile neutropenia during screening. In all cases, the infections were adequately controlled by the start of treatment.

**Table 1:** Patient demographics and baseline characteristics

Characteristic	Value (*N* = 80)
Median age, years (range)	60.0 (34–84)
Sex, *n* (%)	
Male	63 (79)
Female	17 (21)
Race	
White, *n* (%)	72 (90)
Black, *n* (%)	1 (1)
Asian, *n* (%)	1 (1)
Other	3 (4)
Information missing	3 (4)
Ethnicity	
Hispanic or Latino	5 (6)
Not Hispanic or Latino	67 (84)
Not stated	2 (3)
Unknown	6 (8)
Variant HCL, *n* (%)	3 (4)
Splenectomy, *n* (%)	5 (6)
Number of prior systemic therapies, median (range)	3 (2–11)
> 3 prior lines, *n* (%)	39 (49)
Baseline hemoglobin, g/dL, median (range)	11.1 (6.5–16.3)
Baseline neutrophil count, × 10^3^/µL, median (range)	0.81 (0.1–6.2)
Baseline platelet count, × 10^3^/µL, median (range)	68.0 (6.0–350.0)
Prior cancer therapy, *n* (%)	
PNA	80 (100)
Rituximab	60 (75)
BRAF inhibitor	14 (18)
Interferon-α	20 (25)
Other	8 (10)
Unfit for PNA re-treatment, *n* (%)	30 (38)
At risk of infection^a^	20 (25)
Active infection^b^	19 (24)
Patients refractory to PNA,^c^ *n* (%)	39 (49)
PNA monotherapy^d^	29 (36)
PNA + rituximab^e^	15 (19)

*HCL* hairy cell leukemia, *OR* overall response, *PNA* purine nucleoside analog

^a^Patients whose baseline absolute neutrophil count was < 0.5 × 10^3^/µL

^b^Patients whose medical history included a serious infection or febrile neutropenia marked as ‘ongoing’ or ending after the first dose of moxetumomab

^c^Patients whose HCL was refractory to any line of PNA. Note: A patient was counted as having HCL refractory to PNA monotherapy and/or PNA + rituximab

^d^Patients who did not achieve an OR or who achieved an OR lasting < 1 year

^e^Patients who did not achieve an OR or who achieved an OR lasting < 2 years

Fifty patients (63%) completed six cycles of treatment, and 37 patients (46%) completed the study phase, defined as being followed up to Day 181 after the last treatment (irrespective of whether or not they completed treatment). Of the 30 patients (38%) that did not complete six cycles of treatment, 12 had a CR with MRD negativity; 12 discontinued due to AEs; two had disease progression; one died as a result of septic shock that was unrelated to treatment; and three discontinued for other reasons (Additional file 1: Figure S1).

Overall, 43 patients (54%) discontinued the study: Three (4%) withdrew consent, two (3%) were lost to follow up, four (5%) died, and 34 (43%) discontinued for other reasons, mainly due to disease progression or because they started an alternative anti-cancer therapy (Additional file 1: Figure S1). All deaths were considered unrelated to study treatment.

### Efficacy

Disease responses, as assessed by BICR and the investigators for the ITT population, are presented in Table 2. At a median follow-up of 24.6 months (range: 1.2–71.7 months), the durable CR rate (CR with HR > 180 days) was 36% (29 patients; 95% CI, 26–48%). This surpassed the target rate of 28% and was significantly higher than the historical control (lower bound of 95% CI > 13%). Moreover, the CR rate with HR ≥ 360 days was 33% (26 patients; 95% CI 22–44%). Thirty-three patients had CR, 8 of whom of had a spleen size > 13 cm (range: 13.2–16.1 cm); all 8 of these patients remained in HR at 180 days, and 7 of them remained in HR at 360 days.

**Table 2:** Moxetumomab pasudotox was associated with a high response rate and MRD negativity among complete responders

Parameter	Value (*N* = 80), ITT population	
	Blinded independent central review	Investigator assessment
Durable CR [primary endpoint], *n* (%)	29 (36)	39 (49)
95% CI^a^	26, 48	37, 60
CR with HR ≥ 360 days, *n* (%)	26 (33)	36 (45)
95% CI^a^	22, 44	34, 57
Best overall response		
CR, *n* (%)	33 (41)	42 (53)
95% CI^a^	30, 53	41, 64
CR and MRD–, *n* (%)^b^	27 (34)	26 (33)
95% CI^a^	24, 45	22, 44
CR and MRD + , *n* (%)^b^	6 (8)	6 (8)
95% CI^a^	3, 16	3, 16
PR, *n* (%)	27 (34)	21 (26)
Objective response rate [CR or PR], *n* (%)	60 (75)	63 (79)
95% CI^a^	64, 84	68, 87
Median duration of response, months (range)	66.7 (0+ to 66.7)	42.1 (0.0 + to 69.0)
Median duration of CR, months (range)	62.8 (0.1+ to 62.8)	56.6 (0.0 + to 69.0)

CR was defined as clearing of the bone marrow of hairy cells (determined by routine hematoxylin and eosin stain), radiologic resolution of pre-existing lymphadenopathy and/or organomegaly, and HR. PR was defined as ≥ 50% decrease or normalization (< 500/mm^3^) in peripheral blood lymphocyte count, reduction of pre-existing lymphadenopathy and/or organomegaly, and HR or 50% improvement in neutrophils, platelets, and hemoglobin over baseline (without transfusions or growth factors for at least 4 weeks)

*CI* confidence interval, *CR* complete response, *HR* hematologic remission, *IHC* immunohistochemistry, *ITT* intent-to-treat, *MRD* minimal residual disease, *PR* partial response

^a^Two-sided CI was calculated using the exact probability method based on the binomial distribution. ^b^Determined by IHC

In the majority of cases (51 out of 80 patients, 64%), response assessments were concordant between BICR and local settings. Among the 33 BICR-categorized CRs, 29 were also investigator-categorized CRs, including 28 durable CRs; among the 42 investigator-categorized CRs, 13 were not reported as CR by BICR. The discrepancies were due to differences in assessment of bone marrow involvement, technical challenges (e.g., unavailable/unevaluable H&E slides), and incomplete disease assessments by local investigators. Some level of variability is to be expected with low levels of disease (e.g., 1–2% involvement).

**Fig. 1:**
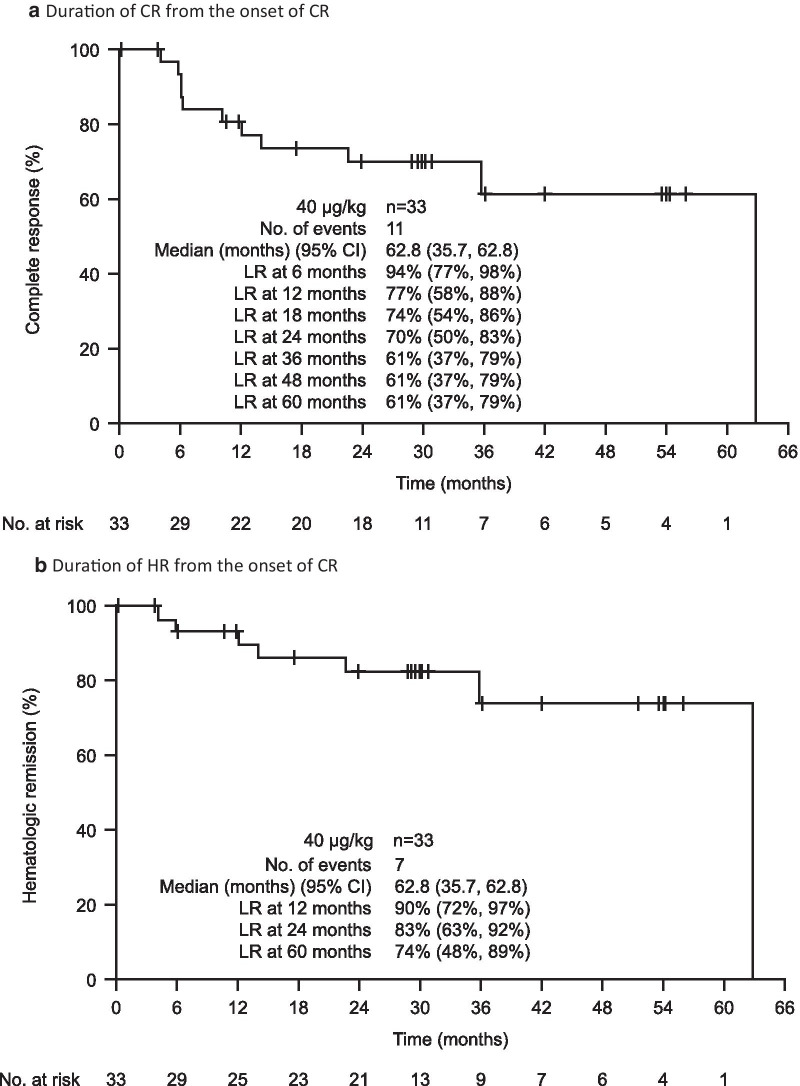

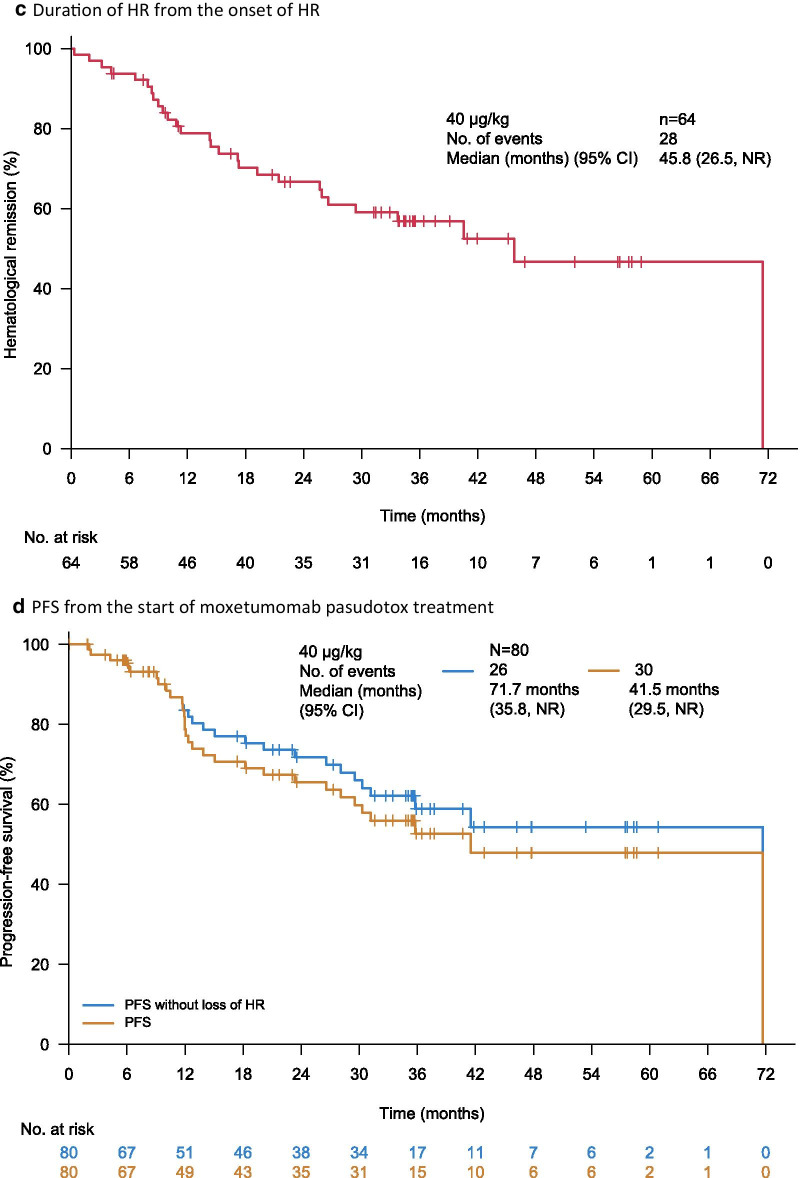
Moxetumomab pasudotox provided durable responses in heavily pre-treated patients. As assessed by BICR in the ITT population, Kaplan–Meier plots of **a** duration of CR and **b** duration of HR, in patients with CR, **c** duration of HR in all patients with HR, and **d** PFS. HR is defined as hemoglobin ≥ 11.0 g/dL, absolute neutrophil count ≥ 1.5 × 10^3^/µL, and platelet count ≥ 100 × 10^3^/µL, without receiving transfusions or growth factors within the preceding 4 weeks of assessment. *BICR* blinded independent central review, *CI* confidence interval, *CR* complete response, *HR* hematologic remission, *ITT* intent-to-treat population, *LR* landmark rate, *NE* not evaluable, *NR* not reported, *OR* objective response, *PFS* progression-free survival

Based on BICR, 60 patients (75%; 95% CI, 64–84%) had an OR, of which 33 (41%; 95% CI, 30–53%) had a CR; both the median CR duration and median HR duration from the onset of CR were 62.8 months (Fig. 1a, b). Landmark rates of CR at 6, 12, 18, 24, 36, 48, and 60 months were 94%, 77%, 74%, 70%, 61%, 61%, and 61%, respectively. The median OR duration was 66.7 months, and the median HR duration from the onset of HR was 45.8 months (Fig. 1c). Finally, median PFS was 41.5 months if a progression event was considered as the loss of any CR criteria; median PFS was longer (71.7 months) if a progression event had to be accompanied by loss of HR (Fig. 1d).

Notably, the median estimates for the duration of response (e.g., CR and HR) were only reached at the last event, when a single patient was at risk. For this reason, the landmark analyses provide a more robust measure of treatment efficacy than the median estimates. Nevertheless, these analyses should be interpreted with caution owing to the small number of patients at the later time points.

**Fig. 2:**
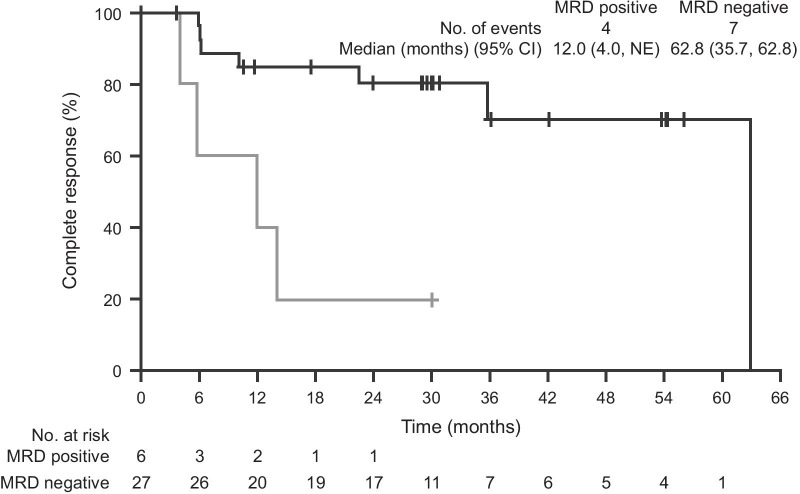
MRD negativity was associated with durable CR. Kaplan–Meier plot for the patients with a complete response in the ITT population, as assessed by BICR (*n* = 33). The starting point of the observation was from the onset of CR and MRD testing. *BICR* blinded independent central review, *CI* confidence interval, *CR* complete response, *ITT* intent-to-treat, *MRD* minimal residual disease, *NE* not evaluable

Overall, 27 (82%) of the 33 patients with a CR also demonstrated MRD negativity (BICR), representing 34% of all patients. Both the median duration of CR (62.8 vs 12.0 months; Fig. 2) and median duration of HR from CR (62.9 [95% CI: NE] vs 12.0 months [95% CI 5.88–NR months]) were longer in MRD-negative patients than in MRD-positive patients.

### Safety

For the primary analysis, safety data were collected up to 30 days after the last dose of moxetumomab pasudotox was administered. SAEs were followed until resolution or death. All patients had completed at least 6 months of post-treatment follow-up by the date of the primary analysis cutoff. Therefore, limited safety data were collected after this date (no new information on SAEs), and safety results in this long-term follow-up analysis remain largely unchanged [29].

Overall, 79 subjects (99%) experienced at least one AE, of which the most common were peripheral edema (39%), nausea (35%), and fatigue (34%) (Additional file 2: Table S1). The most commonly occurring grade 3–4 events were decreased lymphocyte count in 16 patients (20%), anemia in 8 patients (10%), and asymptomatic hypophosphatemia in 8 patients (10%). AEs not reported as related to study treatment led to study discontinuation in 4 patients (5%): glioblastoma, grade 5 pneumonia, grade 1 memory impairment, and grade 4 sepsis syndrome. Secondary malignancies were not a safety issue in this study. There was one report of glioblastoma and multiple myeloma each, and six cases of non-melanoma skin cancers (1 basal cell carcinoma, 2 lipomas, 2 seborrheic keratoses, and 1 squamous cell carcinoma), but these were not reported as related to moxetumomab pasudotox treatment.

The most frequent treatment-related AEs were nausea (22 patients, 28%), peripheral edema (21 patients, 26%), headache (17 patients, 21%), and pyrexia (16 patients, 20%) (Table 3). Treatment-related grade 3–4 AEs were reported in 24 patients (30%) and treatment-related SAEs in 14 (18%). Overall, treatment-related AEs led to study drug discontinuation in 8 patients (10%): hemolytic uremic syndrome (HUS; 4 patients, 5%), capillary leak syndrome (CLS; 2 patients, 3%), and increased blood creatinine (2 patients, 3%). Notably, HUS and CLS events were generally reversible.

**Table 3:** Summary of treatment-related AEs^a^

	Treatment-related AEs of all grades	Grade 3–4 treatment-related AEs
	Patients, *n* (%)	Patients, *n* (%)
Nausea	22 (28)	2 (3)
Edema peripheral	21 (26)	0
Headache	17 (21)	0
Pyrexia	16 (20)	1 (1)
Capillary leak syndrome	7 (9)	2 (3)
Hemolytic uremic syndrome	6 (8)	4 (5)
Lymphocyte count decreased	6 (8)	6 (8)
Anemia	5 (6)	2 (3)
Platelet count decreased	5 (6)	2 (3)
Hypertension	4 (5)	2 (3)
Acute kidney injury	2 (3)	2 (3)
Neutropenia	2 (3)	2 (3)
White blood cell count decreased	2 (3)	2 (3)

*AE* adverse event

^a^Adverse events of any grade with an incidence of at least 20%, as well as events of grade 3 or 4 with an incidence of at least 3%

Key laboratory findings are reported in  Additional file 3: Table S2. All serum creatinine laboratory values stayed within normal limits up to 12 months post-treatment, and there was no decline in renal function over time ( Additional file 4: Figure S2). With a median follow-up of 24.6 months, four deaths were reported: two due to HCL progression and two due to an AE (1 each of pneumonia and septic shock). The fatal pneumonia was reported in a 79-year-old female 30 days after the last administration of study medication. Given the long delay between this event and the last administration of study medication, the event was considered not to be related to moxetumomab pasudotox by the investigator. The septic shock was reported in a 70-year-old male, known to have grade 3 neutropenia at baseline. One hundred and twenty-nine days after starting study medication, this patient was hospitalized for septic shock due to *Staphylococcus* and died 4 days later. In light of the baseline neutropenia and the 4 months of therapy with infectious complications, this event was considered not to be related to moxetumomab pasudotox by the investigator. Therefore, overall no deaths were considered to be related to moxetumomab pasudotox administration.

## Discussion

HCL is a rare malignancy, accounting for approximately 2% of all leukemias. Thus, there is a lack of rigorous and prospective studies evaluating therapies, particularly in the relapsed/refractory setting [30, 31]. To date, this is the largest prospective study to evaluate therapy in the third line and beyond for HCL, demonstrating an unprecedented duration of CR (> 5 years), high OR rate (75%), and long-lasting PFS (median of 41.5 months) in heavily pre-treated patients. Consistent with the primary analysis, the durable CR rate was significantly higher than the CR rate of 13% in the largest rituximab trial, which was used as a historical control for this study [11, 29]. Notably, this is also the first trial to both establish and meet the primary endpoint of durable CR (with HR > 180 days) in heavily pre-treated patients, setting an important standard for assessing the clinical benefit of therapies in future trials.

Unfortunately, studies with long-term follow-up for other late-line HCL treatments are scarce. The majority of rituximab studies have included ≤ 10 patients with heavily pre-treated disease and a follow-up of less than 3 years [9, 10, 12, 20]. Hence, the efficacy of rituximab monotherapy has not been well established in this setting (≥ 2 prior therapies). Perhaps the strongest previous data come from small Phase 2 studies of vemurafenib, ibrutinib (including variant HCL), and vemurafenib plus rituximab in patients who had received a median of ≥ 3 prior therapies [8, 17, 19]. These studies have demonstrated CRs lasting around 2 years, but the largest included only 27 evaluable patients. By contrast, the current study estimated that the probability of CRs lasting at least 5 years is > 60% in patients treated with moxetumomab pasudotox. This finding is promising but should be interpreted with caution owing to the limited number of patients at later time points.

The proportion of patients who achieved a durable CR rate with moxetumomab pasudotox in the long-term follow-up analysis, as determined by BICR, was approximately 20% higher than in the primary analysis (29 patients, 36% vs 24 patients, 30%) [29]. This difference was due to some patients having a follow-up of < 180 days at the date of the primary analysis cutoff.

Beyond the conventional efficacy and safety endpoints evaluated, this pivotal study also examined MRD status (using bone marrow IHC) and its influence on outcomes. Published data suggest that MRD is not routinely assessed in clinical trials, and the few trials that have evaluated MRD status after therapy included a limited number of patients and follow-up [1, 2, 8–19]. For example, preliminary results from a Phase 2 study of vemurafenib plus rituximab demonstrate encouraging MRD-negativity rates (63% at approximately 30 months) but in only 27 patients [19]. In contrast, none of the patients in an Italian study of vemurafenib monotherapy were reported as MRD-negative [8].

This study of moxetumomab pasudotox is the largest trial to prospectively evaluate MRD with long-term follow-up and found a clear association between negative MRD status and durable remission. The majority of complete responders demonstrated MRD negativity (27 patients, 82%) with moxetumomab pasudotox. Both the median duration of CR and duration of HR from CR were approximately fivefold longer in MRD-negative (62.8 months and 62.9 months, respectively) versus MRD-positive patients (12.0 months and 12.0 months, respectively) treated with moxetumomab pasudotox, highlighting the potential clinical relevance of prospective MRD assessments. This long duration of HR from CR in MRD-negative patients is an important finding, given that the reappearance of cytopenias is of greater clinical relevance than the reappearance of leukemic cells. Indeed, median PFS was longer if progression occurred without the loss of HR (71.7 months vs 41.5 months).

In this long-term follow-up, moxetumomab pasudotox treatment continued to demonstrate the manageable safety profile and acceptable tolerability reported in the primary analysis. Relative to PNAs, this safety profile is largely favorable as moxetumomab pasudotox results in less myelotoxicity and immunosuppression and thus does not increase susceptibility to infection [2, 29, 30]. Notably, this study did not evaluate moxetumomab pasudotox in patients with significant comorbidities such as compromised organ function, nor those with active or ongoing infections; all cases of intercurrent infection were adequately controlled by the start of treatment. Thus, careful patient assessment and selection will be necessary prior to treatment with moxetumomab pasudotox.

Infrequently, HUS and CLS can occur with moxetumomab pasudotox treatment and proactive monitoring of patients for the relevant symptoms is important. However, these conditions are both manageable and reversible with best supportive care. Current prevention strategies for HUS include oral hydration and i.v. fluid supplementation (0.5–1 L) before and after each dose, and the oral use of dexamethasone in those with nausea or pyrexia. Serum creatinine levels remained within the normal range 1 year after treatment, and there was no decline in renal function with moxetumomab pasudotox treatment over time.

## Conclusions

Although a larger randomized, controlled trial would ideally be required to confirm the positive effects of moxetumomab pasudotox reported in this open-label trial, patient numbers in this rare condition are small and there are limited treatment options available. Based on the primary analysis [29], which demonstrated promising efficacy and acceptable safety data, moxetumomab pasudotox became the first therapy to receive FDA approval for R/R HCL in September 2018. Here, we show that moxetumomab pasudotox provides an unprecedented rate of deep and durable CR with a manageable safety profile in R/R disease. Moreover, the majority of patients with CR also demonstrate MRD negativity and thus may benefit from improved long-term clinical outcomes. The results of this trial represent an important step forward for patients with R/R HCL, providing a clinically meaningful option following initial treatment with a PNA.

## Supplementary information

**Additional file 1: Figure S1**. Patient disposition. Patient disposition diagram for the 89 patients that were screened. ^a^Informed Consent Form signed. ^b^Completion of protocol treatment is defined as six cycles of therapy. ^c^Completion of study phase is defined as being followed up to Day 181 after the last treatment, regardless of the number of doses of moxetumomab received.

**Additional file 2: Table S1**. Summary of AEs. Summary of treatment-emergent AEs of all grades and grades 3 to 4 in the Safety population. Adverse events of any grade with an incidence of at least 20%, as well as events of grade 3 or 4 with an incidence of at least 3%. AE, adverse event.

**Additional file 3: Table S2**. Summary of changes in laboratory values from baseline. The median changes in hematological laboratory values from baseline are summarized for patients in the Safety population by minimum, maximum, EOT. Baseline indicates assessment prior to first dose. EOT, end of treatment; max, maximum; min, minimum.

**Additional file 4: Figure S2**. Mean serum creatinine over time. Creatinine levels over time (mean +/– SD) from the intent-to-treat population are shown; N = 80.

## Data Availability

The datasets generated and/or analyzed during the current study are available from the corresponding author upon reasonable request.

## Abbreviations

AEs: Adverse events
BICR: Blinded independent central review
CI: Confidence interval
CLS: Capillary leak syndrome
CR: Complete response
CT: Computed tomography
ECOG: Eastern Cooperative Oncology Group
H&E: Hematoxylin and eosin
HCL: Hairy cell leukemia
HR: Hematologic remission
HUS: Hemolytic uremic syndrome
IHC: Immunohistochemistry
ITT: Intent-to-treat
i.v.: Intravenous
MRD: Minimal residual disease
MRI: Magnetic resonance imaging
NE: Not evaluable
OR: Objective response
PCR: Polymerase chain reaction
PFS: Progression-free survival
PNA: Purine nucleoside analogs
R/R: Relapsed/refractory
SAEs: Serious adverse events
