# Supplementary figures and images for: Moxetumomab pasudotox in heavily pre-treated patients with relapsed/refractory hairy cell leukemia (HCL): long-term follow-up from the pivotal trial
Source: J Hematol Oncol. 2021 Feb 24;14:35. doi: 10.1186/s13045-020-01004-y (PMC7905554; doi:10.1186/s13045-020-01004-y)

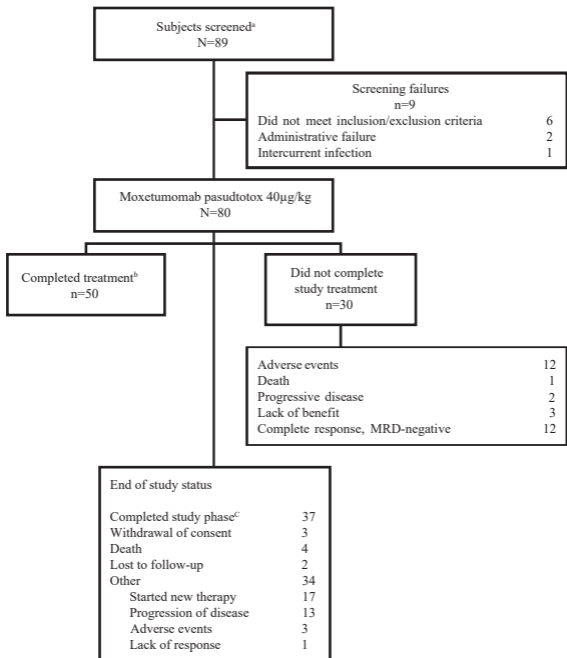

Supplement: Supplementary file 1 — Additional file 1: Figure S1. Patient disposition. Patient disposition diagram for the 89 patients that were screened. aInformed Consent Form signed. bCompletion of protocol treatment is defined as six cycles of therapy. cCompletion of study phase is defined as being followed up to Day 181 after the last treatment, regardless of the number of doses of moxetumomab received. [file 13045_2020_1004_MOESM1_ESM.pdf]

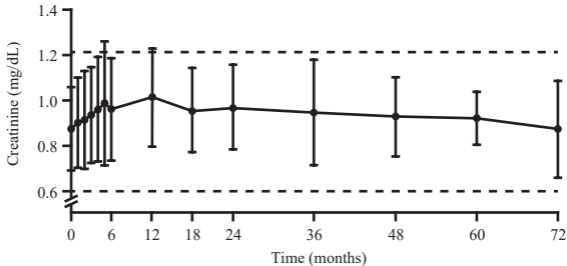

No. of patients: 80

55

30

52

41

35

13

4

4

Supplement: Supplementary file 4 — Additional file 4: Figure S2. Mean serum creatinine over time. Creatinine levels over time (mean +/– SD) from the intent-to-treat population are shown; N = 80. [file 13045_2020_1004_MOESM4_ESM.pdf]
