# Supplementary material for: Moxetumomab pasudotox in heavily pre-treated patients with relapsed/refractory hairy cell leukemia (HCL): long-term follow-up from the pivotal trial
Source: J Hematol Oncol. 2021 Feb 24;14:35. doi: 10.1186/s13045-020-01004-y (PMC7905554; doi:10.1186/s13045-020-01004-y)
Supplement: Supplementary file 2 — Additional file 2: Table S1. Summary of AEs. Summary of treatment-emergent AEs of all grades and grades 3 to 4 in the Safety population. Adverse events of any grade with an incidence of at least 20%, as well as events of grade 3 or 4 with an incidence of at least 3%. AE, adverse event. [file 13045_2020_1004_MOESM2_ESM.docx]

**Table S1. Summary of AEs^a^**

|  | **AE of all grades** | **Grades 3–4 AE** |
| --- | --- | --- |
|  | **Patients, n (%)** | **Patients, n (%)** |
| Edema peripheral | 31 (39%) | 0 |
| Nausea | 28 (35%) | 2 (3%) |
| Fatigue | 27 (34%) | 0 |
| Headache | 26 (33%) | 0 |
| Pyrexia | 25 (31%) | 1 (1%) |
| Hypocalcemia | 19 (24%) | 0 |
| Hypophosphatemia | 19 (24%) | 8 (10%) |
| Constipation | 18 (23%) | 0 |
| Anemia | 17 (21%) | 8 (10%) |
| Diarrhea | 17 (21%) | 0 |
| Alanine aminotransferase increased | 17 (21%) | 1 (1%) |
| Lymphocyte count decreased | 16 (20%) | 16 (20%) |
| Hypoalbuminemia | 16 (20%) | 0 |
| Hypokalemia | 13 (16%) | 2 (3%) |
| Hypertension | 12 (15%) | 6 (8%) |
| Platelet count decreased | 9 (11%) | 5 (6%) |
| Hyponatremia | 9 (11%) | 2 (3%) |
| White blood cell count decreased | 8 (10%) | 7 (9%) |
| Capillary leak syndrome | 7 (9%) | 2 (3%) |
| Upper respiratory infection | 7 (9%) | 2 (3%) |
| Hemolytic uremic syndrome | 6 (8%) | 4 (5%) |
| Neutrophil count decreased | 6 (8%) | 5 (6%) |
| Febrile neutropenia | 5 (6%) | 4 (5%) |
| Neutropenia | 4 (5%) | 4 (5%) |
| Hypoxia | 4 (5%) | 2 (3%) |
| Lung infection | 3 (4%) | 2 (3%) |
| Acute kidney injury | 3 (4%) | 2 (3%) |
| Erysipelas | 2 (3%) | 2 (3%) |

**^a^**Adverse events of any grade with an incidence of at least 20%, as well as events of grade 3 or 4 with an incidence of at least 3%.

AE, adverse event.
