# Supplementary material for: Moxetumomab pasudotox in heavily pre-treated patients with relapsed/refractory hairy cell leukemia (HCL): long-term follow-up from the pivotal trial
Source: J Hematol Oncol. 2021 Feb 24;14:35. doi: 10.1186/s13045-020-01004-y (PMC7905554; doi:10.1186/s13045-020-01004-y)
Supplement: Supplementary file 3 — Additional file 3: Table S2. Summary of changes in laboratory values from baseline. The median changes in hematological laboratory values from baseline are summarized for patients in the Safety population by minimum, maximum, EOT. Baseline indicates assessment prior to first dose. EOT, end of treatment; max, maximum; min, minimum. [file 13045_2020_1004_MOESM3_ESM.docx]

Additional file 3: Table S2. Summary of changes in laboratory values from baseline.^a^

| **Parameter** | **Baseline^a^**  **N=80** | **Baseline median** | **Visit or Condition** | **Median** | **Change from Baseline** |
| --- | --- | --- | --- | --- | --- |
|  | **n** |  |  |  | **Median (min–max)** |
| Hemoglobin (g/dL) | 80 | 11·10 | Minimum on treatment | 10·20 | -0·90 (-4·7–2·2) |
|  |  |  | Maximum on treatment | 13·90 | 2·40 (-0·8–6·3) |
|  |  |  | End of treatment | 13·30 | 1·90 (-2·1–6·3) |
| Platelets (10^3^/µL) | 80 | 68·80 | Minimum on treatment | 63·00 | 0·0 (-87·0–74·0) |
|  |  |  | Maximum on treatment | 247·00 | 159·0 (6·0–451·0) |
|  |  |  | End of treatment | 97·00 | 97·0 (-41·0–279·0) |
| Neutrophils (10^3^/µL) | 80 | 0·81 | Minimum on treatment | 0·55 | -0·19 (-5·6–0·5) |
|  |  |  | Maximum on treatment | 4·60 | 3·53 (-0·9–14·7) |
|  |  |  | End of treatment | 2·70 | 1·68 (-1·3–5·9) |
| CD4+ T cells (/µL) | 64 | 350·0 | Minimum post baseline | 222·0 | -50·0 (-1283–108) |
|  |  |  | End of treatment | 390·0 | 86·5 (-167–870) |
|  |  |  | Day 181 post EOT | 375·0 | 103·0 (-255–528) |

The median changes in hematological laboratory values from baseline are summarized for patients in the Safety population by minimum, maximum, EOT. ^a^Baseline indicates assessment prior to first dose.
EOT, end of treatment; max, maximum; min, minimum.
